# Efficacy and safety of intravitreal faricimab for neovascular age-related macular degeneration: a systematic review and meta-analysis

**DOI:** 10.1038/s41598-024-52942-3

**Published:** 2024-01-30

**Authors:** Wei-Ting Yen, Chen-Shu Wu, Chang-Hao Yang, Yi-Hao Chen, Cho-Hao Lee, Cherng-Ru Hsu

**Affiliations:** 1 Department of Ophthalmology, Tri-Service General Hospital, National Defense Medical Center, Taipei, Taiwan; 2 Department of Internal Medicine, Tri-Service General Hospital, National Defense Medical Center, Taipei, Taiwan; 3 Department of Ophthalmology, National Taiwan University Hospital, Taipei, Taiwan; 4 Department of Ophthalmology, National Taiwan University College of Medicine, Taipei, Taiwan; 5 Division of Hematology and Oncology Medicine, Department of Internal Medicine, Tri-Service General Hospital, National Defense Medical Center, No. 325, Sec. 2, Chenggong Rd., Neihu Dist., Taipei City, 114 Taiwan; 6 Department of Ophthalmology, Show Chwan Memorial Hospital, No. 542, Sec. 1, Zhongshan Rd., Changhua City, 500 Changhua County Taiwan

**Keywords:** Outcomes research, Drug therapy, Retinal diseases

## Abstract

We conducted a systematic review and meta-analysis to evaluate the visual, anatomical, and safety outcomes of the intravitreal faricimab, a novel vascular endothelial growth factor (VEGF)/angiopoietin-2 (Ang-2) bispecific agent, in neovascular age-related macular degeneration (nAMD) patients. The follow-up times in the included studies ranged from a minimum of 36 weeks to a maximum of 52 weeks. EMBASE, Ovid-Medline, Cochrane Central Register of Controlled Trials (CENTRAL), Web of Science, Scopus, the WHO ICTRP, ClinicalTrial.gov, the EU Clinical Trials Register, and Chinese Clinical Trial Registry (ChiCTR) were searched (The last literature search was performed on August 17, 2023) for randomized controlled trials (RCTs) comparing faricimab with control groups for neovascular age-related macular degeneration (nAMD). The risk of bias for eligible RCTs was independently assessed using the Cochrane Risk of Bias Tool by two authors (W.-T.Y. and C.-S.W.). The meta-analysis was conducted using Review Manager 5.4 software. The mean best corrected visual acuity (BCVA), central subfield thickness (CST), total choroidal neovascularization (CNV) area, and total lesion leakage were analyzed as continuous variables and the outcome measurements were reported as the weighted mean difference (WMD) with a 95% confidence interval (CI). The ocular adverse events and ocular serious adverse events were analyzed as dichotomous variables and the outcome measurements were analyzed as the odds ratios (ORs) with a 95% CI. Random-effects model was used in our study for all outcome synthesizing due to different clinical characteristics. Four RCTs with 1,486 patients were eligible for quantitative analysis. There was no statistically significant difference between intravitreal faricimab and anti-VEGF in BCVA [weighted mean difference (WMD) = 0.47; 95% CI: (− 0.17, 1.11)]. The intravitreal faricimab group showed numerically lower CST [WMD =  − 5.96; 95% CI = (− 7.11, − 4.82)], total CNV area [WMD =  − 0.49; 95% CI = (− 0.68, − 0.30)], and total lesion leakage [WMD =  − 0.88; 95% CI = (− 1.08, − 0.69)] after intravitreal therapy compared with the intravitreal anti-VEGF group. There were no statistically significant differences between intravitreal faricimab and anti-VEGF in ocular adverse events (AEs) [pooled odds ratio (OR) = 1.10; 95% CI = (0.81, 1.49)] and serious adverse events (SAEs) [pooled OR = 0.84; 95% CI = (0.37, 1.90)]. The intravitreal bispecific anti-VEGF/angiopoietin 2 (Ang2) antibody faricimab with a extended injection interval was non-inferior to first-line anti-VEGF agents in BCVA. It was safe and had better anatomical recovery. Large, well-designed RCTs are needed to explore the potential benefit of extended faricimab for nAMD. This systematic review was registered in the International Prospective Register of Systematic Reviews (PROSPERO) database (CRD42022327450).

## Introduction

Neovascular age-related macular degeneration (nAMD) remains a main cause of irreversible blindness in the elderly over 60 years of age in developed countries^1–4^. Despite evidence showing that antioxidant vitamin and mineral supplements may delay the progression of age-related macular degeneration (AMD)^5,6^, development of nAMD is sometimes inevitable. Although several anti-vascular endothelial growth factor (VEGF) agents have been proven to be effective in improving vision outcomes in nAMD patients^7^, development of a more effective therapeutic agent with fewer adverse effects remains warranted.

Overwhelming evidence has demonstrated that VEGF plays a pivotal role in abnormal retinal angiogenesis^8^, which makes VEGF a potential target for medical therapies. Faricimab, a novel VEGF/angiopoietin-2 (Ang-2) bispecific agent, has gained the attention of researchers. It simultaneously inhibits VEGF and Ang2 signaling and leads to better vascular stability and less retinal inflammation compared to monotherapy with anti-VEGF^9^. This unique characteristic of faricimab makes it potentially preferable because it provides better outcomes for visual acuity and has an extended treatment interval, which may minimize the treatment burden for a patient requiring intravitreal injection therapy^10^.

Several network meta-analyses have reported the comparative efficacy and safety of anti-VEGFs for retinal vascular diseases. In the study by Tricco et al., it was found that ranibizumab, bevacizumab, aflibercept, and brolucizumab had statistically significant advantages over conbercept regarding the proportion of nAMD patients who experienced moderate vision gain^11^. Virgili et al. highlighted that, for individuals with diabetic macular edema (DME), aflibercept presented certain benefits over ranibizumab and bevacizumab after one year, both visually and anatomically^12^. Conversely, Sangroongruangsri and colleagues emphasized that, among patients with retinal vein occlusion (RVO), bevacizumab, ranibizumab, and aflibercept outperformed sham injections regarding BCVA enhancement and central macular thickness decline, with each maintaining a commendable safety profile^13^. Zhao et al. demonstrated that treat-and-extend intravitreal ranibizumab, compared to a fixed dose, could produce a better visual outcome in nAMD^14^. Notably, faricimab has not been incorporated into network meta-analysis comparisons in previous research. Faricimab, a bispecific antibody targeting both VEGF and Ang-2, has emerged as a potentially safe and promising solution in the management of nAMD. Despite the increasing attention it has received, comprehensive evidence assessing its effectiveness remains sparse. To address this, we conducted a rigorous systematic review by delving into multiple databases and clinical trial registries, aiming to provide a meta-analysis centered on evaluating the visual and anatomical outcomes of nAMD patients treated with faricimab.

## Methods

### Literature search

The current study followed the guidelines of the Preferred Reporting Items for Systematic Reviews and Meta-analyses (PRISMA)^15^. EMBASE, Ovid-Medline, Cochrane Central Register of Controlled Trials (CENTRAL), Web of Science, and Scopus databases were searched systematically to identify relevant studies. The last literature search was performed on August 17, 2023 by W.-T.Y. The WHO ICTRP, ClinicalTrial.gov, the EU Clinical Trials Register, and Chinese Clinical Trial Registry (ChiCTR) were also searched for ongoing clinical trials. We primarily used the following keywords for our search: maculopathy, macular degeneration, macular neovascularization, age-related macular degeneration, and faricimab. Detailed search syntax can be found in eTable 1 of the supplement. This systematic review was registered in the International Prospective Register of Systematic Reviews (PROSPERO) database (CRD42022327450).

### Inclusion/exclusion criteria

The inclusion of studies was based on the following criteria: (1) randomized controlled trial (RCT), (2) adult patients with neovascular age-related macular degeneration (nAMD), (3) use of intravitreal faricimab as an experimental arm, (4) comparison of faricimab versus anti-VEGF agents, and (5) reported at least one clinical outcome, such as best corrected visual acuity (BCVA), central subfield thickness (CST), or adverse events. The exclusion criteria were: (1) phase I clinical trials, (2) studies involving choroidal neovascularization due to causes other than age-related macular degeneration (AMD). Whether the articles were to be included was independently screened by W.-T.Y. and C.-S.W. using the EndNote X9 version.

### Data extraction and quality assessment

Data from the included studies were extracted by two authors, W.-T.Y. and C.-S.W. In cases of disagreement, C.-R.H. was consulted to resolve the issue. The risk of bias for eligible RCTs was independently assessed using the Cochrane Risk of Bias Tool by both W.-T.Y. and C.-S.W.^16^. If there were disputes regarding the risk of bias, C.-R.H. made the final decision, with reference to the guidance provided in the Cochrane Reviewer’s Handbook^17^.

### Data synthesis and analysis

The meta-analysis was conducted using Review Manager 5.4 software. The mean best corrected visual acuity (BCVA), central subfield thickness (CST), total choroidal neovascularization (CNV) area, and total lesion leakage were analyzed as continuous variables and the outcome measurements were reported as the weighted mean difference (WMD) with a 95% confidence interval (CI). The proportion of gaining ≥ 0, 5, 10, and 15 Early Treatment of Diabetic Retinopathy Study (ETDRS) letters, proportion with BCVA 20/40 or better, proportion with BCVA 20/200 or worse, and ocular adverse events were analyzed as dichotomous variables and the outcome measurements were analyzed as the odds ratios (ORs) with a 95% confidence interval (CI). A *p* value < 0.05 was regarded as statistically significant. Heterogeneity of the included studies was assessed with I square (I^2^) and Q test analyses, and *p* < 0.10 and I^2^ > 50% were regarded as significant heterogeneity. Sensitivity analysis was used to assess robustness of the synthesized results. Funnel plots were used to assess the publication bias. Subgroup analysis was used to investigate significant heterogeneity based on specific characteristics, such as types of anti-VEGF. We used the random-effects model in our study for all outcome synthesizing due to different clinical characteristics. The inverse variance method and Mantel–Haenszel method were used for synthesizing continuous variables and dichotomous variables, respectively. The GRADE system was used to rate the certainty of evidence^18^.

### Ethics approval

Ethics committee approval was not required, as our study was completed using publicly available published data.

### Provenance and peer review

Not commissioned; externally peer reviewed.

## Results

### Literature search

**Figure 1:**
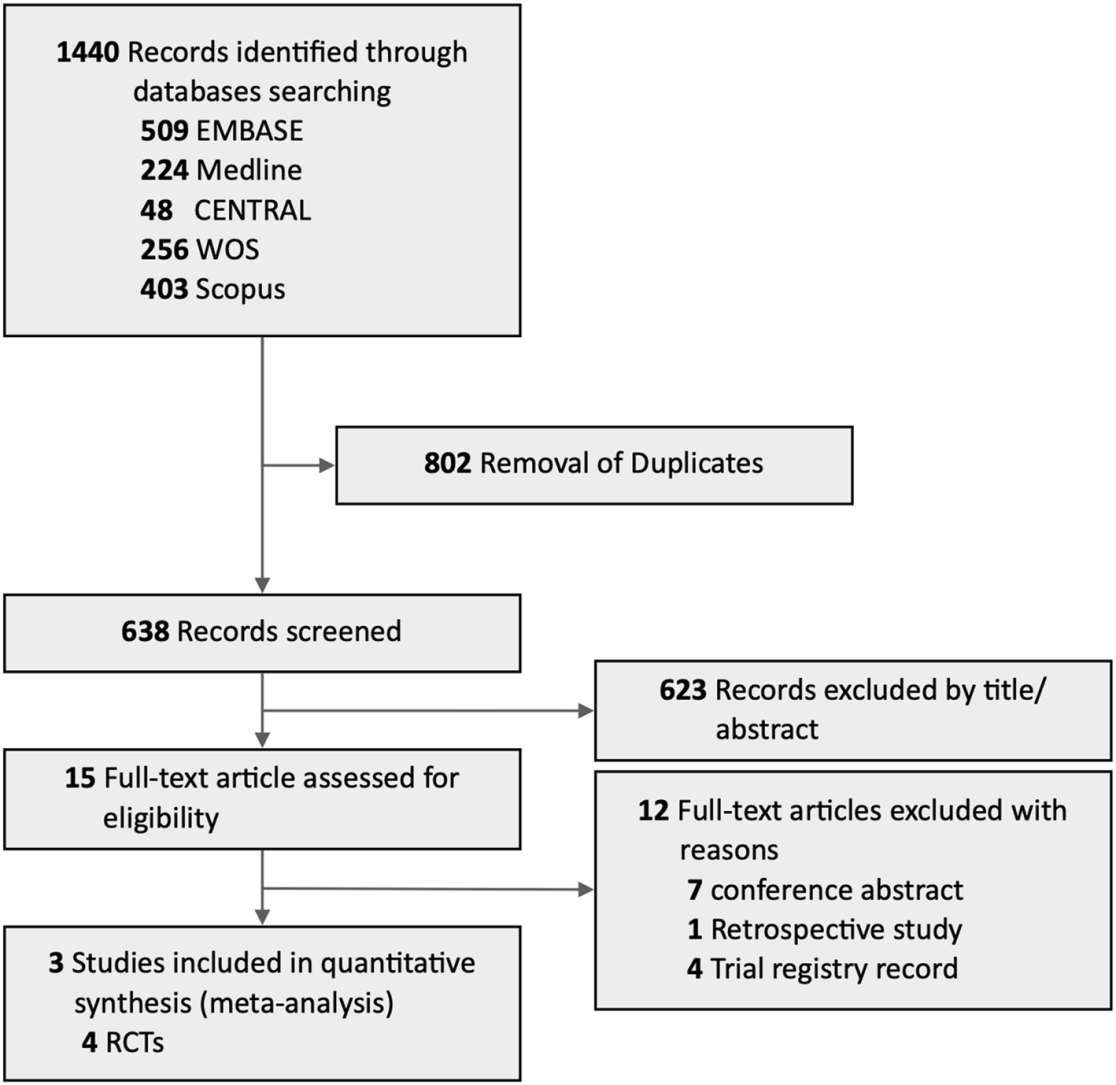
PRISMA flow diagram of the literature search process for eligible randomized controlled trials.

The process of literature search is summarized in Fig. 1. The initial search in EMBASE, Ovid-Medline, CENTRAL, Web of Science, and Scopus databases yielded 1440 relevant references. Of these, 802 were excluded due to duplication. Due to irrelevant titles or abstracts, an additional 623 refences were excluded. Thereafter, the remaining 15 full-text articles were reviewed for eligibility. Three studies, including four RCTs, were finally included in the quantitative meta-analysis. The detailed search strategies, processes, and results are presented in eTable 1.

### Characteristics of the eligible trials

The characteristics of the four included RCTs are summarized in Table 1. All of the studies enrolled adult patients older than 50 years with a diagnosis of nAMD. In the AVENUE study (NCT02484690), 273 patients were randomized to five arms (arms A–E) and were followed for a period of up to 36 weeks^19^. Among these arms, the patients in arm A received 0.5 mg ranibizumab every four weeks as an active comparator. We included arm D (6.0 mg faricimab every four weeks up to week 12, followed by 6.0 mg every eight weeks) for data analysis because they had the most extended intravitreal injection interval. In the STAIRWAY study (NCT03038880), 76 patients were randomized into three groups and were followed for a period of up to 52 weeks^20^. Sixteen patients were assigned to receive ranibizumab (0.5 mg) every four weeks as an active comparator. We included 31 patients who received faricimab (6.0 mg) every 16 weeks as an experimental group for outcome analysis. In the TENAYA (NCT03823287) and LUCERNE (NCT03823300) studies, 671 and 658 patients were randomized into two groups, respectively, and were followed for a period of up to 48 weeks^21^. The patients who were assigned to the control group received aflibercept 2.0 mg every four weeks up to week 8 (three injections), followed by fixed 8-week dosing to the study end. The patients who were assigned to the experimental group received faricimab 6.0 mg up to every 16 weeks based on disease activity. BCVA (ETDRS letter score) was regarded as the primary endpoint in all four RCTs. These four RCTs all reported: (1) patients gaining ≥ 15 EDTRS letters, (2) BCVA 20/40 or better, (3) CST, (4) total lesion area, and (5) Total lesion leakage as the functional and anatomical outcomes. Ocular adverse events and ocular serious adverse events were all documented in the safety profiles of the four RCTs.

**Table 1:** Characteristics of Included Random Controlled Trials Regarding the Efficacy and Safety Outcomes of intravitreal Faricimab and Anti-VEGFS Treatment.

Source	Study design	Study population	Intervention				Follow-up, weeks	Main outcomes
			Experimental	Sample size	Controlled	Sample size		
Sahni, et al.^19^ (AVENUE)	Phase 2 RCT	Treatment-naïve Choroidal Neovascularization (CNV) Secondary to Age-Related Macular Degeneration	Arm B: Faricimab, 1.5 mg every 4 weeks	Arm B: 47	Arm A:	68	36	Mean BCVA (ETDRS) change (compared to Arm A):
			Arm C: Faricimab, 6.0 mg every 4 weeks	Arm C: 42	Ranibizumab, 0.5 mg every 4 weeks			Arm B: 1.6 (80% CI, − 1.6 to 4.7) letters
			Arm D: Faricimab, 6.0 mg every 4 weeks up to week 12, followed by 6 mg every 8 weeks	Arm D: 47				Arm C: − 1.6 (80% CI, − 4.9 to 1.7) letters
			Arm E: Ranibizumab 0.5 mg + Faricimab 6.0 mg every 4 weeks	Arm E 69				Arm D: − 1.5 (80% CI, − 4.6 to 1.6) letters
								Arm E: − 1.7 (80% CI, − 3.8 to 0.4) letters
Khanani et al.^20^	Phase 2 RCT	Treatment-naive CNV secondary to AMD (nAMD)	Arm B: Faricimab, 6.0 mg every 12 weeks	Arm B: 29	Arm A:	16	52	Mean BCVA (ETDRS) change (compared to Arm A):
(STAIRWAY)			Arm C: Faricimab, 6.0 mg every 16 weeks	Arm C: 31	0.5 mg Ranibizumab every 4 weeks			Arm B: 0.5 (80% CI, –4.3, 5.3) letters
								Arm C: 1.8 (80% CI, –2.7, 6.4) letters
Heier et al.^21^	Phase 3 RCT	Treatment-naïve choroidal neovascularization (CNV) secondary to age-related macular degeneration (nAMD) in the study eye	Faricimab 6.0 mg up to every 16 weeks, based on activity assessments	331	Aflibercept 2.0 mg every 4 weeks up to week 8, followed by fixed 8-week dosing to study end	327	48	Mean BCVA (ETDRS) change:
(LUCERNE)								Faricimab group: 6·6 letters (95% CI 5·3 to 7·8)
								Aflibercept: 6·6 letters (5·3 to 7·8)
								(treatment difference 0·0 letters [95% CI –1·7 to 1·8])
Heier, et al.^21^	Phase 3 RCT	Treatment-naïve choroidal neovascularization (CNV) secondary to age-related macular degeneration (nAMD) in the study eye	Faricimab 6.0 mg up to every 16 weeks, based on activity assessments	334	Aflibercept 2·0 mg every 4 weeks up to week 8, followed by fixed 8-week dosing to study end	337	48	Mean BCVA (ETDRS) change:
(TENAYA)								Faricimab group: 5·8 letters (95% CI 4·6 to 7·1)
								Aflibercept group: 5·1 letters (3·9 to 6·4) (treatment difference 0·7 letters [95% CI − 1·1 to 2·5])

### Quality of the included trials

The quality of the four included RCTs was rigorously assessed through the Cochrane Risk of Bias Tool, as delineated in eFigure 1. The trials generally exhibited a low risk of bias across the main six domains. However, it is notable that in the 'other bias' domain, the risk was categorized as 'unclear' due to the funding received from pharmaceutical companies, suggesting a potential conflict of interest.

### Efficacy analysis

#### BCVA

**Figure 2:**
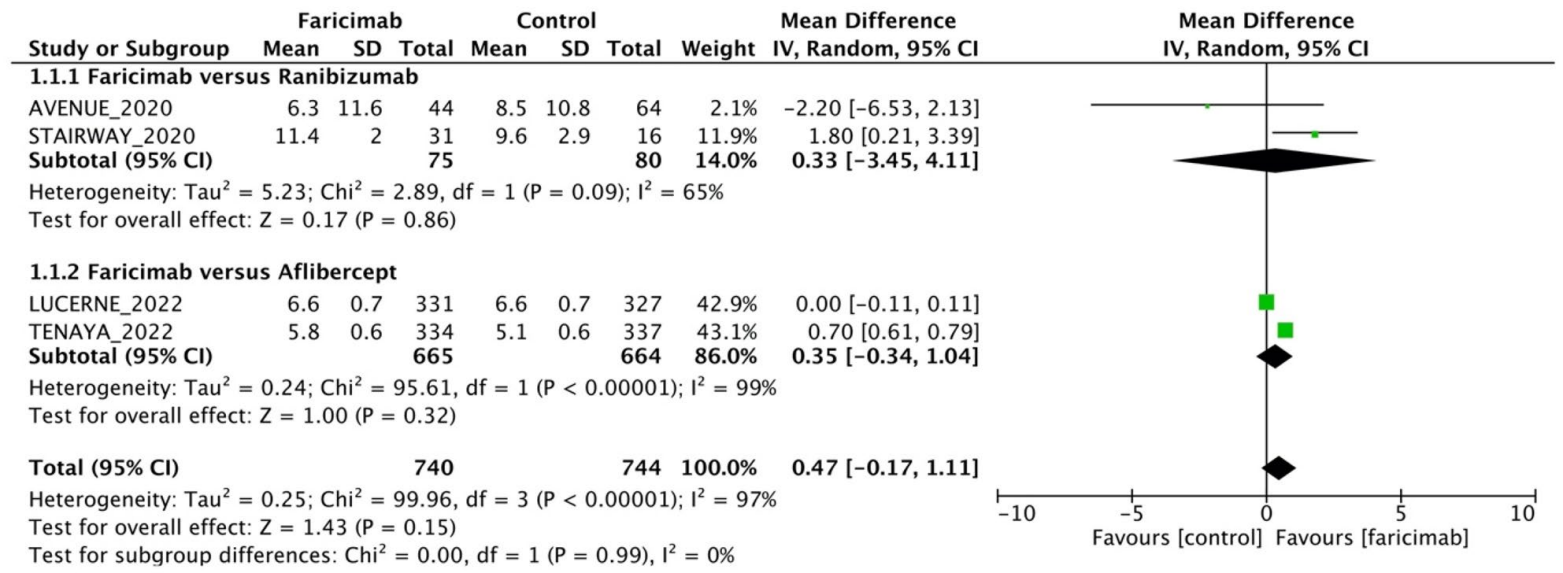
Mean difference in best-corrected visual acuity (ETDRS letters) between intravitreal faricimab and control groups.

A total of 1,484 eyes from 4 RCTs were pooled to estimate the WMD of BCVA (Fig. 2). All four studies included in the analysis assessed the BCVA via ETDRS letters. The intravitreal faricimab group showed numerically higher BCVA after intravitreal therapy compared with the intravitreal anti-VEGF group [WMD = 0.47; 95% CI = (− 0.17, 1.11)], but the difference in BCVA between the intravitreal faricimab group and anti-VEGF group was not statistically significant. Our meta-analysis further demonstrated no differences in the proportion of patients who gained15, 10, 5, and 0 EDTRS letters or more BCVA [pooled OR = 1.03; 95% CI = (0.76, 1.39), pooled OR = 1.22; 95% CI = (0.96, 1.54), pooled OR = 1.11; 95% CI = (0.82, 1.52), and pooled OR = 1.08; 95% CI = (0.82, 1.42), respectively; eFig. 2]. Comparable outcomes were also observed in the proportion of patients with BCVA of 20/40 or better and with BCVA 20/200 or worse between the faricimab and the control groups [pooled OR = 1.15; 95% CI = (0.80, 1.65)] and pooled OR = 1.02; 95% CI = (0.67, 1.54), respectively; eFigs. 3, 4]. Funnel plot for BCVA was reported in eFigure 5.

### Subgroup analysis

#### Faricimab versus ranibizumab

**Figure 3:**
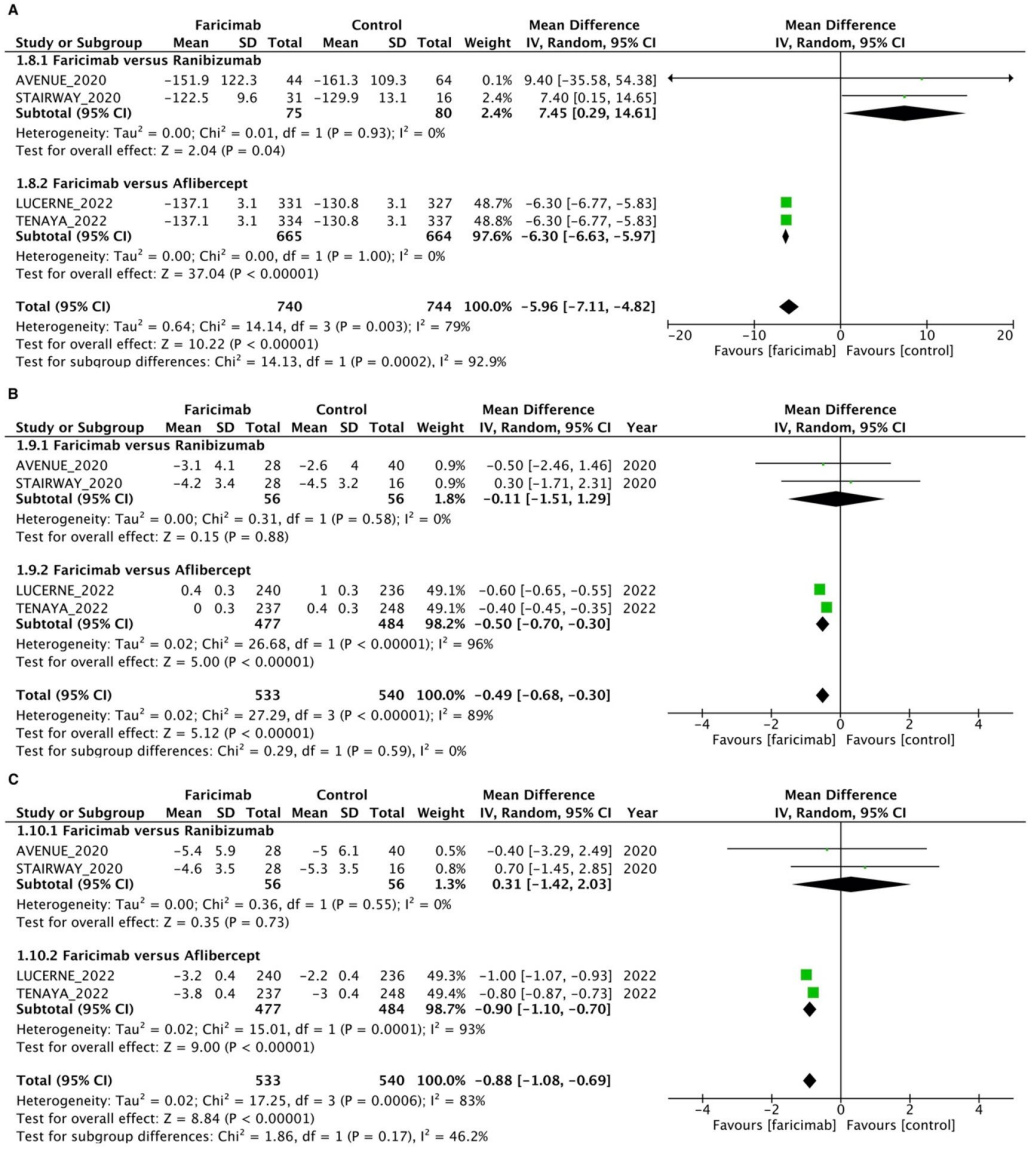
Mean difference in anatomical outcomes between intravitreal faricimab and control groups. (**A**) Central subfield thickness (µm). (**B**) Total CNV area (mm^2^). (**C**). Total lesion leakage (mm^2^).

For faricimab versus ranibizumab subgroup analysis, a total of 155 eyes from two RCTs were pooled to estimate the WMD of BCVA between faricimab and ranibizumab (Fig. 2). All two studies included in the analysis assessed the BCVA via ETDRS letters. The intravitreal faricimab group showed numerically better BCVA after intravitreal therapy compared with the intravitreal ranibizumab group [WMD = 0.33; 95% CI = (− 3.45, 4.11)], but the difference in BCVA between the intravitreal faricimab group and the ranibizumab group was not statistically significant. A total of 155 eyes from two RCTs were pooled to estimate the WMD of CST between faricimab and ranibizumab (Fig. 3A). The intravitreal faricimab group showed numerically higher CST after intravitreal therapy compared with the ranibizumab group [WMD = 7.45; 95% CI = (0.29, 14.61])], and the difference in CST between the intravitreal faricimab group and the ranibizumab group was statistically significant. The intravitreal faricimab group showed numerically lower CNV area after intravitreal therapy compared with the ranibizumab group [WMD =  − 0.11; 95% CI = (− 1.51, 1.29)], and the difference in total CNV area between the intravitreal faricimab group and the ranibizumab group was statistically insignificant (Fig. 3B). The intravitreal faricimab group showed numerically higher total lesion leakage after intravitreal therapy compared with the ranibizumab group [WMD = 0.31; 95% CI = (− 1.42, 2.03)], and the difference in total lesion leakage between the intravitreal faricimab group and the ranibizumab group was statistically insignificant (Fig. 3C).

### Faricimab versus aflibercept

For faricimab versus aflibercept subgroup analysis, a total of 1,329 eyes from two RCTs were pooled to estimate the WMD of BCVA between faricimab and aflibercept (Fig. 2). All two studies included in the analysis assessed BCVA via ETDRS letters. The intravitreal faricimab group showed numerically greater BCVA after intravitreal therapy compared with the intravitreal aflibercept group [WMD = 0.35; 95% CI = (− 0.34, 1.04)], but the difference in BCVA between the intravitreal faricimab group and the aflibercept group was not statistically significant. A total of 1,329 eyes from two RCTs were pooled to estimate the WMD of CST between faricimab and aflibercept (Fig. 3A). The intravitreal faricimab group showed numerically lower CST after intravitreal therapy compared with the aflibercept group [WMD =  − 6.3; 95% CI = (− 6.63, − 5.97)], and the difference in CST between the intravitreal faricimab group and the aflibercept group was statistically significant. The intravitreal faricimab group showed a numerically smaller total CNV area after intravitreal therapy compared with the aflibercept group [WMD =  − 0.50; 95% CI = (− 0.70, − 0.30)], and the difference in total CNV area between the intravitreal faricimab group and the aflibercept group was statistically significant (Fig. 3B). The intravitreal faricimab group showed numerically lower total lesion leakage after intravitreal therapy compared with the aflibercept group [WMD =  − 0.90; 95% CI = (− 1.10, − 0.70)], and the difference in total lesion leakage between the intravitreal faricimab group and the aflibercept group was statistically significant (Fig. 3C).

### Safety analysis

**Figure 4:**
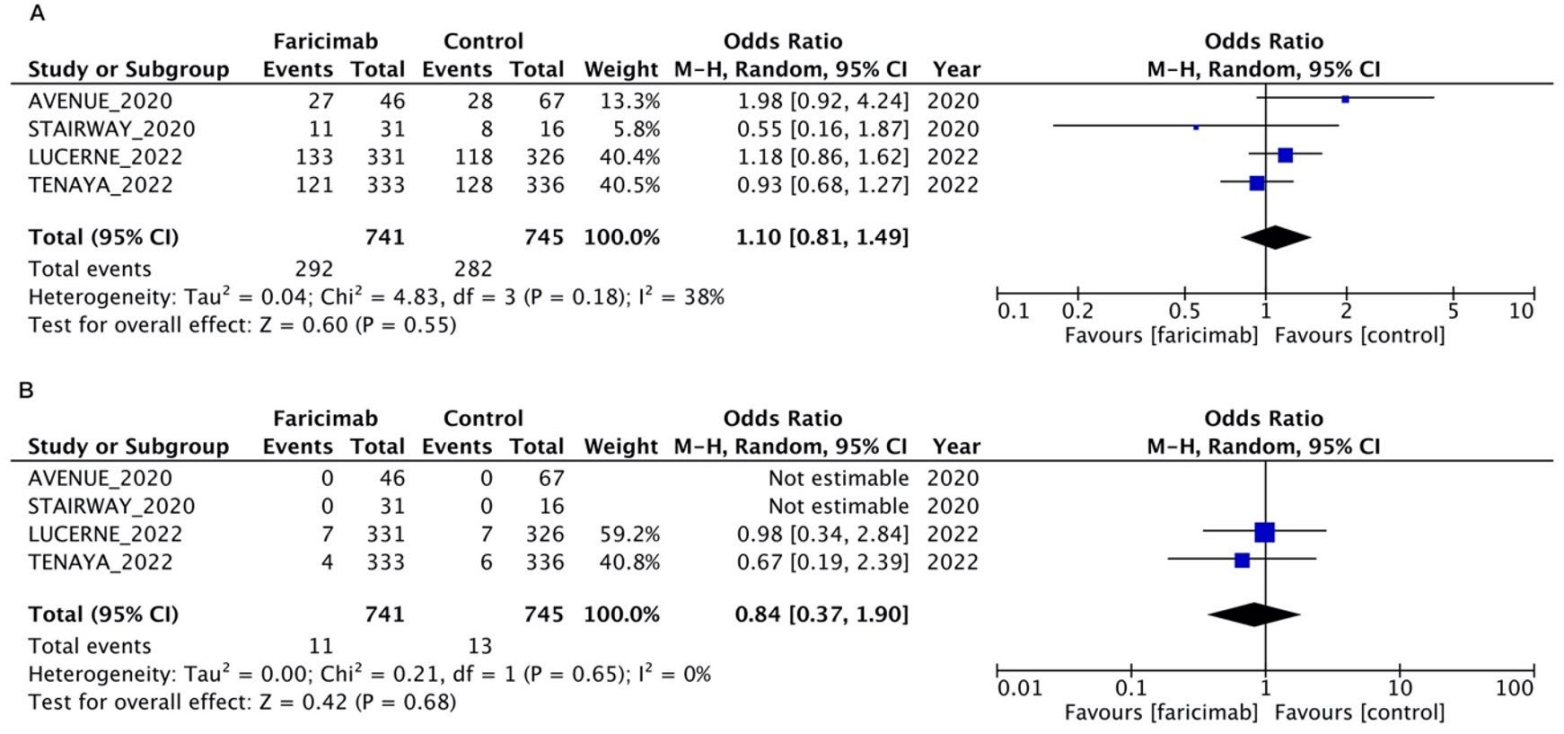
Odds ratio for ocular adverse events in intravitreal faricimab and control groups. (**A**) Ocular adverse events. (**B**). Ocular serious adverse events.

A total of 1,486 eyes from four RCTs was pooled to estimate the odds ratios of ocular adverse events (Fig. 4A) and ocular serious adverse events (Fig. 4B). There were no significant differences between the intravitreal faricimab group and anti-VEGF group in the incidence of ocular adverse events [pooled OR = 1.10; 95% CI = (0.81, 1.49)] and ocular serious adverse events [pooled OR = 0.84; 95% CI = (0.37, 1.90)].

### Quality of evidence

The results of the quality of evidence (GRADE system) analysis are summarized in eTable 2.

## Discussion

In our study, we demonstrated the mean CST change from baseline was significantly more prominent in treatment with extended intervals of the novel humanized bispecific antibody faricimab than treatment with aflibercept, whereas no difference was identified in visual outcomes in both the ranibizumab and aflibercept subgroup analyses. Regarding the safety issue, our results showed faricimab was not inferior to ranibizumab or aflibercept. Faricimab potentiated a durable effect with fewer injections, which decreased the clinical burdens of nAMD patients.

A similar functional outcome with superior anatomical outcome with faricimab in comparison with aflibercept was found for treatment efficacy in this meta-analysis. While there was significant improvement in retinal thickness, this did not correspond to definite restoration of visual function. These results may reflect the heterogeneity of baseline characteristics in nAMD patients. The CST decrement due to structural impairment such as geographical atrophy or ellipsoid disruption may be associated with a poor visual prognosis, while retinal thinning due to decreased vessel leakage may correlate with visual gain^22^. Active CNV lesions resulting in increments of CST accompanied by sub retinal fluid (SRF) may be tolerable and correlate with stable or improved visual acuity^23^. However, new scar tissue formation, persistent intraretinal fluid (IRF), or sub-retinal pigment epithelium (RPE) fluid accumulation seemed to negatively affect visual acuity^22^. Also, fluctuation in CST may be associated with a higher likelihood of fluid persistence with less improvement in visual acuity^24^. Although discrepancy between the trials regarding the treatment endpoint assessment, a mean difference of at least one line of visual acuity gain and 100 µm CST decrease than baseline with nearly half of the faricimab-treated patients on extended fixed treatment intervals of 16 weeks demonstrated faricimab’s sustained dosing potential^20,21^.

Quantification of disease activity represented by angiographic behavior have shown area reductions compared to baselines after anti-VEGF treatment^25^. Our meta-analysis showed superior efficacy of faricimab for anatomical aspects as the mean change in total area of CNV lesions and total area of leakage with an estimated 0.49 mm^2^ and 0.88 mm^2^ significant involution, respectively. The anti-inflammatory, antiangiogenic, and vascular homoeostasis function of Ang-2 might theoretically improve efficacy in nAMD compared to therapies with anti-VEGF pathway alone.

While patients diagnosed with polypoidal choroidal vasculopathy (PCV) and retinal angiomatous proliferation (RAP) lesions were included in TENAYA and LUCERNE studies^21^, both were excluded in the AVENUE and STAIRWAY studies^19,20^. Previous research found inconsistencies in visual acuity improvement in PCV and RAP compared to the typical nAMD^26,27^, yet the visual outcomes did not show significant differences following anti-VEGF therapy in our analysis. Further research may be required to investigate the treatment effect on nAMD subtypes.

The safety of intravitreal anti-VEGF therapy in contemporary treatment is well-established; that is, ocular adverse events such as infection or inflammation-related situations remain a primary concern^28^. Our analysis yielded a consistently low incidence of adverse events with faricimab and aflibercept or ranibizumab. Notably, one case treated with faricimab suffered from endophthalmitis that was attributed to the injection procedure instead of being drug induced. No cases of retinal detachment were reported. In addition, most nAMD patients are older in age with varying degrees of disability in real-world settings. They thereby require a more intensive regimen causing systemic exposure and this should be carefully investigated. Reibaldi et al. suggested that frequent injections had no significant influence on mortality^29^. Similarly, our results showed comparable severe adverse events for faricimab and aflibercept without unexpected safety issues.

Despite the latest and comprehensive evaluation of our meta-analysis, there were some limitations in our study. First, high heterogeneity exists between the included RCTs, which were partially addressed by our subgroup analyses. Secondly, the small number and limited follow-up times of the eligible studies that were included resulted in favorable clinical outcomes for faricimab efficacy; however, evidence from the real-world and the long-term follow-up to test the non-inferiority in BCVA, morphology outcomes, and adverse events are lacking. Third, direct comparison of the extended durability of faricimab is absent as a consequence of the either fixed 8-week or 4-week dosing administration of the comparator in the studies.

In addition to the currently published RCTs, the AVONELLE-X study (NCT04777201) is a recruiting phase 3 clinical trial that is evaluating the long-term safety and tolerability of intravitreal faricimab for nAMD^30^. Although our meta-analysis provided the first evidence analysis of faricimab for nAMD, further large, well-designed clinical studies employing the T&E (treat and extend) strategy are warranted to determine the best therapeutic approach.

### Supplementary Information

Supplementary Information.

## Data Availability

All data included in the current study was extracted from the electronic databases and published articles.
